# The Effect of SARS-CoV-2 Virus Infection on the Course of Atopic Dermatitis in Patients

**DOI:** 10.3390/medicina57060521

**Published:** 2021-05-22

**Authors:** Martyna Miodońska, Agnieszka Bogacz, Magdalena Mróz, Szymon Mućka, Andrzej Bożek

**Affiliations:** Clinical Department of Internal Disease, Dermatology and Allergology in Zabrze, Medical University of Silesia, 40-055 Katowice, Poland; martynamiodonska298@gmail.com (M.M.); agnieszka.anna.bogacz@gmail.com (A.B.); mrozm@onet.pl (M.M.); szymuska44@gmail.com (S.M.)

**Keywords:** COVID-19, atopic dermatitis, immunosuppression

## Abstract

*Background and Objectives*: Atopic dermatitis (AD) is a disease with a complex pathophysiology involving immune-mediated reactions that lead to skin lesions that are typically localized and recurrent. Following the outbreak of the COVID-19 (coronavirus disease 2019) pandemic, attempting to assess the impact of SARS-CoV-2 infection on diseases caused by complex immune mechanisms has become important. The aim of this study was to assess the impact of SARS-CoV-2 infection on the course of AD, including immunosuppressive therapy, in patients with a severe form of the disease. *Materials and Methods*: A retrospective analysis of 21 adults aged 18 to 52 years with AD diagnosed with COVID-19, including patients requiring hospitalization, was performed. *Results*: During SARS-CoV-2 infection, temporary exacerbation of skin lesions and/or skin pruritus was observed in nine (43%) patients but without the need for systemic treatment intervention. Patients with severe AD who received immunosuppressive therapy most often manifested mild exacerbation of skin symptoms. The skin condition improved in three patients. There was no significant effect of disease severity on the risk of severe COVID-19 (HR = 0.45; 95% CI: 0.32–0.65). *Conclusions*: The course of atopic dermatitis during SARS-CoV-2 infection may be different from the severity of its symptoms due to the lack of a significant influence. The immunosuppressive treatment used in patients with severe AD did not significantly affect the course of SARS-CoV-2 infection.

## 1. Introduction

Atopic dermatitis (AD) is a disease that has been increasingly affecting society in recent years [1]. The current prevalence of AD in children is estimated at 15–30% of the population; in adults, it is estimated at 2–10% [2]. Depending on the country, the severe form of the disease can affect 12–21% of AD cases [3,4].

The pathophysiology of AD is complex and includes many factors: genetically determined atopy, disturbed skin epidermal barrier with water loss, dysregulation of the cellular immune response, and environmental factors [5,6]. Th2 cytokines are associated with the early stages of the disease, whereas increased expression of Th1 cytokines appears to promote the transition of AD to chronic disease. Th2 cytokines also activate the production of IgE antibodies by B lymphocytes, which is responsible for the atopic nature of dermatosis [7,8,9,10,11,12].

COVID-19 (coronavirus disease 2019) is an acute infectious disease of the respiratory system caused by infection with the SARS-CoV-2 virus. The first case was recorded in November 2019 in the city of Wuhan, China, during the series of cases that started the pandemic. On 11 March 2021, the WHO listed almost 118 million cases worldwide [13]. Symptoms of SARS-CoV-2 infection concern not only the respiratory system but also the digestive system and the skin. The broad spectrum of the clinical picture observed in the course of COVID-19 is largely associated with comorbidities and conditions modifying immunity, which may worsen the course of infection [14]. Data on the influence of immunosuppressants, including those used in AD, on the course of SARS-CoV-2 infection are not clear [15,16,17]. The use of glucocorticoids may predispose patients to a more severe course of COVID-19 [15,18,19]. On the other hand, it was not observed that biological drug therapy also used in AD and psoriasis had similar effects [15,16,18,20,21,22,23,24,25,26].

The objective of the study was to present the potential effect of SARS-CoV-2 infection on AD of various severities. In addition, an attempt was made to verify the hypothesis that the course of infection could have been previously treated with immunosuppressive drugs used in the abovementioned patients.

## 2. Materials and Methods

This study presents a retrospective analysis of 21 patients with AD treated in the Department of Internal Diseases, Dermatology and Allergology, including the COVID subunit, in Zabrze from May 2020 to April 2021.

The diagnosis of AD was based on the criteria of Hanifin and Rajka. The severity of the disease was calculated on the basis of the SCORAD scale (mild: SCORAD < 30; moderate: SCORAD 40–60; severe: SCORAD > 60). The presence of SARS-CoV-2 infection was confirmed simultaneously by positive PCR results and the presence of a diagnosis of U07.1 (assigned to the diagnosis of symptomatic COVID-19 disease confirmed by a laboratory test).

The group of patients with AD hospitalized due to COVID-19 (*n* = 14) was compared with the group of randomly selected patients with SARS-CoV-2 infection without skin diseases and other associated diseases (*n* = 17) to assess the course of viral infection. These groups were selected in terms of the gender and age of the respondents.

### 2.1. RT-PCR Diagnostics

The SARS-CoV-2 RT-PCR test is a real-time reverse transcription and polymerase chain reaction for the qualitative detection of SARS-CoV-2 nucleic acids in the upper and lower respiratory tract involving samples taken from persons suspected of being infected with COVID-19. Medical staff obtained samples from patients by taking swabs from the throat and atria of the nose. Nucleic acids were extracted from the samples using the MagNA Pure 96 system (Roche, Basel, Switzerland). A real-time targeted RT-PCR assay of the RdRp/Hel SARS-CoV-2 gene was performed using the Quanti Nova Probe RT-PCR kit (QIAGEN, Hilden, Germany).

### 2.2. Statistics

Statistica 8.1 (SoftPols, Krakow, Poland) was used for statistical analysis. Student’s unpaired *t*-test was used for parametric tests involving data with normal distributions, and the Fischer exact test was used for nonparametric tests. The risk of odds (HR) was calculated with a confidence interval of 95% to assess the possible impact of AD stage (assessed according to the SCORAD scale) on the risk of a severe course of SARS-CoV-2 infection (hospitalization). A *p* value of < 0.05 was considered statistically significant.

## 3. Results

The characteristics of the patients are presented in Table 1. The course of AD during SARS-CoV-2 infection varied. No significant worsening of AD, expressed by the need to increase or add systemic immunosuppressive therapy (glucocorticosteroids, cyclosporine, or methotrexate) was observed in any of the patients.

**Figure 1:**
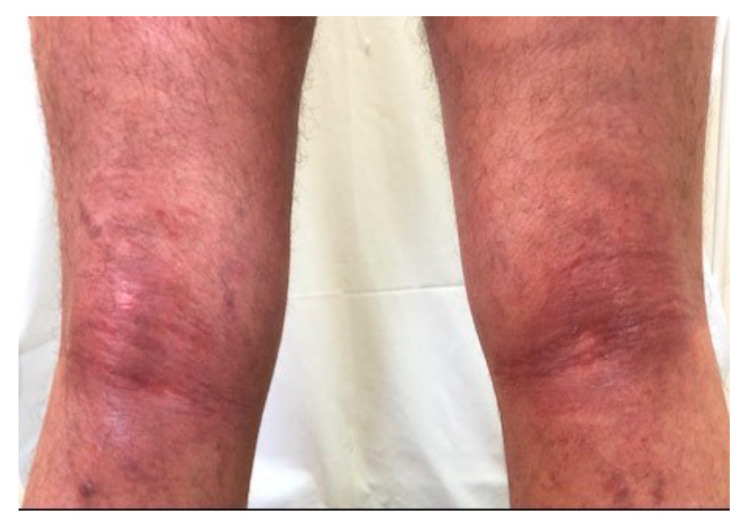
A patient with intensification of skin lesions during COVID-19 infection.

**Figure 2:**
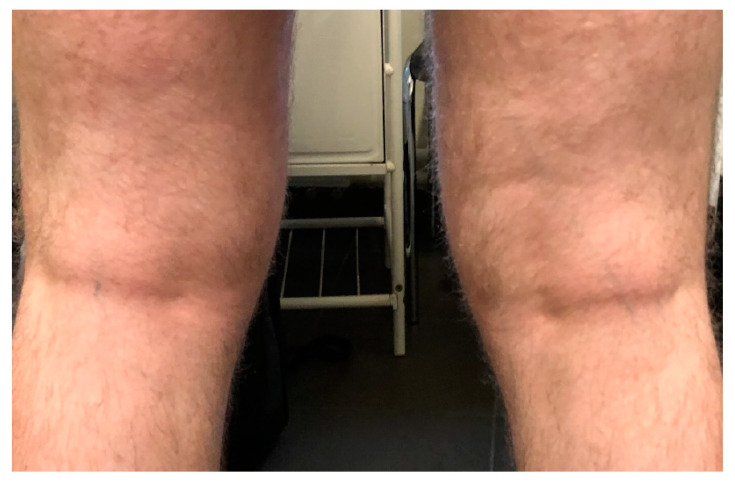
2 weeks after the disease.

Among seven (33%) patients with mild SARS-CoV-2 infection (home treatment), AD mildly worsened in three cases (mean SCORAD change by 15 points). In one patient with severe AD, a significant improvement was observed after infection, which was maintained throughout the entire follow-up and allowed for a reduction of ciclosporin by 50% of the daily dose (Figure 1 and Figure 2). No significant changes in the course of the disease were observed in the remaining patients, including two patients with severe disease.

Despite the need for hospitalization in 14 (67%) of the subjects due to the deterioration of respiratory function in the course of COVID-19, none of the described patients, including those with severe AD on immunosuppressive treatment, had a very severe form of COVID-19 requiring mechanical ventilation. The course of SARS-CoV-2 infection in patients without AD did not differ significantly from that of other hospitalized patients in terms of the duration of stay, treatment, or prognosis. While no deaths were recorded, in the latter, temporary mechanical ventilation was required in three cases.

Data on patients’ hospitalization and the course of AD are presented in Table 2.

There was no significant effect of disease severity on the risk of severe COVID-19 (HR = 0.45, 95% CI: 0.32–0.65).

One month after the end of COVID-19 treatment, no significant changes in the course of AD were observed in 18 (86%) patients compared to the pre-SARS-CoV-2 assessment. Two patients, including one with severe disease, showed an improvement in the reduction of the SCORAD score by more than 30 points. In one patient, the disease worsened from mild (SCORAD = 21) to moderate (SCORAD = 49).

## 4. Discussion

The impact of SARS-CoV-2 infection on the health status of patients suffering from chronic inflammatory diseases remains a constant topic of research. Observations have revealed that AD does not predispose patients to the more severe course of COVID-19 [15,27]. Additionally, in our group of patients, no such relationship was observed. It seems that local immune changes in the upper and lower respiratory tracts are more important in the development of infection, including viral infection, than the systemic immune response [28]. On the other hand, respiratory infections or allergens may exacerbate or complicate the course of inflammatory diseases, including atopic dermatitis [28,29]. In nine of the described cases, there was a temporary exacerbation of AD secondary to infection with SARS-CoV-2. In one patient, the deterioration of the skin condition was of a long-term nature, but it is difficult to associate it with the same type of infection. On the other hand, in patients with more severe AD who used regular systemic glucocorticosteroids, there was no exacerbation of AD. Differences in dermatological therapy could have an impact on the potential exacerbation of AD symptoms during and after infection with SARS-CoV-2, which has also been described by other authors [28,29].

Immunosuppressive and immunomodulatory drugs as well as biological drugs are commonly used in the treatment of many skin diseases, such as atopic dermatitis or psoriasis [30]. However, the use of these drugs is associated with an increased risk of many infections. In the era of a pandemic, many dermatological associations recommend suspending immunosuppressive treatment until the symptoms of COVID-19 have disappeared [31,32]. However, the final decision should be made on the basis of an individual patient’s risk–benefit assessment. It seems that therapy with systemic glucocorticoids may increase the risk of COVID-19 [15] due to the wide immunosuppressive effect of this group of drugs [33]. Therefore, the influence of systemic steroid therapy in the form of prednisone at a dose of 10 or 20 mg in two described patients with COVID-19 infection cannot be excluded.

It is worth emphasizing that the studies published to date do not indicate an increased risk of SARS-CoV-2 infection in patients using biological drugs for psoriasis and atopic dermatitis [16,17,20,23,27]. In light of the ongoing SARS-CoV-2 pandemic, two meta-analyses of the incidence of upper respiratory tract infections (other than COVID-19) during treatment with dupilumab showed that, in controlled studies, there was no increase in systemic infections with antibody therapy [34]. The authors suggest that biological treatment should not be suspended during COVID-19 [25], as a treatment interruption could significantly exacerbate the underlying disease and result in hospitalization [17]. In the only patient treated with dupilumab, the course of SARS-CoV-2 infection was mild, and treatment was continued.

The described patients were hospitalized due to COVID-19 and treated with cyclosporine and methotrexate. Both cyclosporine and methotrexate are associated with a lower rate of infection than systemic corticosteroid therapy and are therefore preferred. However, their impact on susceptibility to SARS-CoV-2 infection and the severity of COVID-19 is unknown. Our observations also do not allow us to clearly assess their impact on the course of SARS-CoV-2 infection.

## 5. Conclusions

The course of atopic dermatitis during SARS-CoV-2 infection may vary, ranging from temporary deterioration to a lack of significant changes. It appears that the baseline stage of AD and the applied immunosuppressive therapy did not significantly affect the course of SARS-CoV-2 infection.

## Figures and Tables

**Table 1:** Characteristics of atopic dermatitis (AD) patients before and during coronavirus disease 2019 (COVID-19) infection.

	Before COVID-19 *n* = 21	During COVID-19 *n* = 21
mean age of patient ± *SD*	37 ± 8	37 ± 8
women	5 (24%)	5 (24%)
severity of AD		
mild (SCORAD < 30)	6 (29%)	3 (14%)
moderate (SCORAD = 40–60)	8 (38%)	11 (52%)
heavy (SCORAD > 60)	7 (33%)	8 (38%)
AD IgE dependent	21 (100%)	21 (100%)
mean value of IgE in serum	1650 kU/L ± 1098 kU/L	1540 kU/L ± 1214kU/L
atopic asthma	2 (10%)	2 (10%)
allergic rhinitis and/or conjunctivitis	6 (29%)	6 (29%)
other concomitant diseases	3 (14%)	5 (24%)
treatment against COVID-19		
antihistamines	18 (86%)	19 (90%)
topical glucocorticosteroids	9 (43%)	13 (62%)
calcineurin inhibitors topically		
	10 (48%)	14 (67%)
systemic glucocorticosteroid	5 (24%) add prednisone: 12.5 mg ± 2.5 mg	9 * (43%)
	4 (19%) add 175 mg ± 50 mg	add 24.5 ± 10 mg
cyclosporine	2 (10%) add 12.5 mg ± 5 mg	4 (19%) add 175 mg ± 50 mg
methotrexate	1 (5%)	2 (10%) add 12.5 mg ± 5 mg
dupilumab		1 (5%)

Legend: add—average daily dose; *—some patients received glucocorticosteroids.

**Table 2:** Comparison of patients with AD to the control group of patients requiring hospitalization due to COVID-19.

Hospitalization for COVID-19	Patients with AD *n* = 14	Control Group *n* = 17	*p*
mean age of patient ± *SD*	47 ± 11	50 ± 8	ns
women	6	7	ns
mean number of days of hospitalization ± *SD*	14 ± 4	16 ± 7	ns
mean O_2_ saturation on admission	88.7 ± 1.3	86.9 ± 2.4	ns
passive oxygen therapy	14 (100%)	13 (76%)	<0.05
mechanical ventilation	0	3 (18%)	<0.05
Treatment	10 (71%)	13 (76%)	ns
remdesivir	8 (57%)	12 (71%)	ns
antibiotics	14 (100%)	16 (94%)	ns
heparin	1 (7%)	4 (23%)	<0.05
plasma of convalescents dexametazon	3 (21%)	5 (29%)	ns
ibuprofen	5 (35%)	7 (41%)	ns
course of AD		-	-
no significant changes compared to the time before infection	6 (14%)		
worsening of itching of the skin	4 (19%)		
new skin changes	3 (21%)		
exacerbation of existing skin changes	2 (14%)		
reduction of skin lesions	2 (14%)		
improvement lasting more than 4 weeks	7 (50%)		
deterioration lasting more than 4 weeks	1 (7%)		

Legend: *SD*—standard deviation; ns—not significant.

## Data Availability

Data supporting results are available in corresponding author upon request.
